# Treatment of idiopathic facial paralysis (Bell’s Palsy) and secondary facial paralysis with extracellular vesicles: a pilot safety study

**DOI:** 10.1186/s12883-023-03400-6

**Published:** 2023-09-28

**Authors:** Paul A. Dreschnack, Ina Belshaku

**Affiliations:** Elmsford, USA

**Keywords:** Extracellular vesicles, Idiopathic facial paralysis (Bell’s Palsy), Secondary facial paralysis, Case report, House-Brackmann scale, Facial Disability Index, ExoFlo™

## Abstract

**Background:**

Paralysis of the facial nerve (CN VII) is one of the most debilitating issues that any patient can encounter. Bell’s palsy is the most commonly seen mononeuropathy. Although usually self-limited, symptomatology can persist for decades in persistent cases. The non-surgical alternative therapies discussed in this study are successful without reconstruction and are regenerative.

**Objective and design:**

We sought to determine a safe new treatment could be developed to restore facial nerve function using extracellular vesicles (EVs) in patients who have been unable to return to normal under a variety of conditions. We performed a pilot safety study of 7 patients with idiopathic and secondary facial paralysis to determine if any functional restoration was possible. Each patient had symptomology for varying periods of time, with diverse House-Brackmann scores. They were all treated with the same protocol of extracellular vesicles (EVs) over a 4-week period of time and were evaluated both before and after treatment.

**Case presentations:**

All patients in this study received treatment by their private physicians prior to entering the study. A record review was completed, with independent physical examinations. House-Brackmann scores and Facial Disability Indices were obtained prior to, and after completing the study. EVs were injected into the area of the main trunk of the facial nerve on the affected side, and an intravenous drip of EVs on visits during weeks 1, 2, and 4.

**Conclusions:**

All seven patients enrolled in the study improved with this treatment protocol. After the second week of treatment, we saw a progression of independent motion of the affected eyelid, brow motion, and commissure. Although all patients began at different House-Brackman starting points, almost all ended at the same endpoint on the scale over the same period of time – four weeks. No adverse effects were encountered.

Clearly, the duration of the treatment protocol needs to be longer than one month. The pathomechanism is still unknown. But it appears that the mechanism is reversible. At last, these patients can have hope.

**Trial registration:**

The Institute of Regenerative and Cellular Medicine IRB approval number: IRCM-2021–304.

## Introduction

A successful treatment of a sentinel case of Bell’s Palsy was reported in 2020, utilizing adipose-derived stem cells [1]. This pilot safety study was planned as a continuation of that report using extracellular vesicles (EVs) for the treatment of facial palsy. The primary objective was to demonstrate a safety profile utilizing EVs for a successful improvement without surgery. Additionally, we wanted to see if any improvement could be achieved toward the ultimate goal of total facial nerve functional restoration.

## Background

The motor nucleus of the pons gives rise to the facial nerve (VII). As it passes through the petrous portion of the temporal bone, it exits the skull through the stylomastoid foramen. Extracranially, the five branches innervate the facial muscles, and the intracranial branches provide sensation to the anterior 2/3 of the tongue, as well as the parasympathetic innervation to the salivary glands, the lachrymal glands, palatine nerves, the stapedius, and the nose.

Paralysis can involve any or all of the extra- or intracranial components. Typically, we witness epiphora or a dry eye, inability to close one eye, drooping of the oral commissure, salivary incompetence, facial tingling, fasciculations of facial muscles, hyperacusis, taste disturbances and the inability to elevate an eyebrow.

The appearance changes drastically, and although the physiological changes can be severe, the social and psychological components are very profound, if not devastating. Many patients report embarrassment due to a breakdown in nonverbal communication. Patients state that if they cannot smile, people treat them “differently”. More specifically, it is assumed that you are anti-social to some extent.

Facial paralysis affects approximately 40,000 people a year in the United States. Traditionally, patients are treated with oral steroids and anti-viral medications within the first three days. Those who are unresponsive to these protocols proceed to attempt alternative modalities, such as acupuncture, botulinum toxin, facial exercises, laser treatment and herbal medicine. There are numerous reports in the literature of successful outcomes [2–7]. Some go on to have facial reconstruction. Unfortunately, many go on with their lives, without the hope of ever having a normally functioning face again.

Emergency room visits are the first stop to rule out cerebrovascular accident. If the workup is unremarkable, they are discharged home with a diagnosis of Bell’s palsy. The etiology is investigated further in some cases but often is not. Not all facial paralysis cases have Bell’s palsy. Some have secondary facial paralysis.

Extracellular vesicles (EVs) are a new frontier in the field of cellular medicine EVs are produced in the endosomal interior of eukaryotic cells. They contain messenger RNA, micro-RNA, cytokines and other proteins in bio-active compounds that decrease inflammation, mediate immune responses to pathogens and tumors via intracellular signaling. The molecular “package” contained in the EV determines its function in the body.

## Methods

In this pilot safety study, we examined seven patients with facial paralysis present for variable lengths of time and reasons, who were not pregnant and have not had reconstructive facial surgery to correct the paralysis. All study procedures and protocols were approved in advance by an independent review board (The Institute of Regenerative and Cellular Medicine). The IRB approval number: is IRCM-2021–304.

Imaging was performed, if not recently completed by their private physician, or reviewed on each patient, along with routine lab analysis, and independent medical screening examinations.

## Participants

All patients had extensive workups by their private physicians at the time of the onset of their symptoms. Many experienced failed therapies and sought supportive care of symptomatology. Patients receiving botulinum toxin, for example, for epiphora ceased therapy prior to entering the program. All other modalities were similarly held for the duration of the study. All persons gave their informed consent prior to their inclusion in the study.

## Case presentation

### Patient #1

**Fig. 1:**
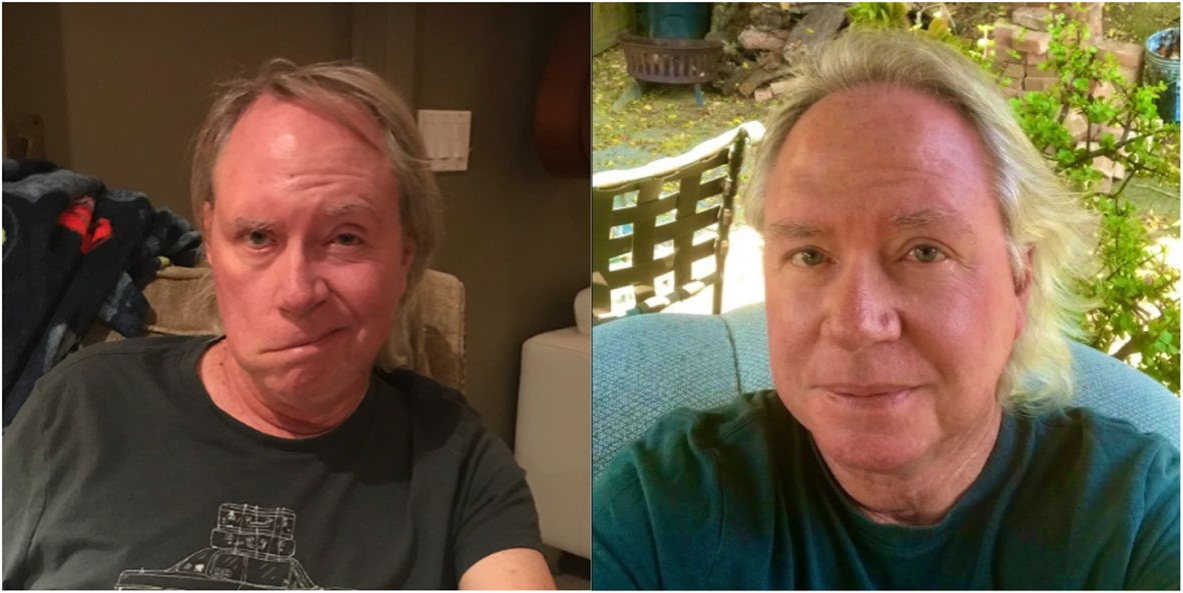
Patient #1. Two years after the onset of right facial paralysis, post acoustic neuroma surgery. Photo one month after completion of extracellular therapy (right)

A 61-year-old male had an acoustic neuroma removed from his right side 2 years ago. His postop recovery was uneventful for twenty-four hours. Then he spontaneously developed an acute onset of right facial paralysis. He was treated with gabapentin, potassium, and diuretics without any improvement. Radiological studies were unremarkable. Mild to moderate symptoms of depression were experienced. (Fig. 1).

### Patient #2

A 38-year-old female developed sudden onset of left-sided facial paralysis in July 2018 when she was 32 weeks pregnant. She was treated with 10 days of prednisone and valaciclovir unsuccessfully. After treatment with massage, physical therapy, and acupuncture, she had a slight improvement nine months later in that she could blink her affected left eye. Radiological studies were unremarkable. Her symptoms persisted for 4 years prior to entering the study. Severe symptoms of depression were experienced.

### Patient #3

A 54-year-old male basketball player developed a spontaneous Bell’s Palsy while touring in Asia. He developed spontaneous acute left-sided facial nerve paralysis. He was treated with acupuncture with unremarkable results. He has had Bell’s Palsy for 10 years with no improvement. MRI of the facial nerve was normal. Mild to moderate symptoms of depression were experienced.

### Patient #4

**Fig. 2:**
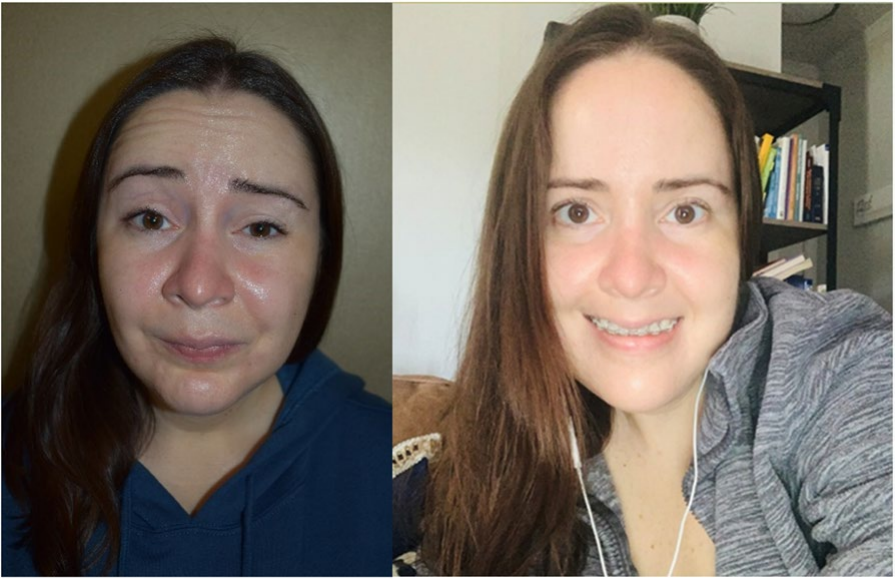
Patient # 4. Left-sided facial paralysis post-TMJ surgery for three years. Post EV treatment (right) for one month

A 32-year-old female with a history of a narrow palate, and an open bite with a receded chin had a LeFort I osteotomy 13 years ago. She developed bilateral condylar reabsorption and had TMJ replacement 3 years prior. She awoke in the recovery room with bilateral facial paralysis. The right side recovered quickly, but the left-sided facial paralysis persisted. She displayed limited progress with facial exercises and steroids. She had facial paralysis with synkinesis for 3 years. She had a history of psoriatic arthritis. Radiological studies were unremarkable for facial nerve pathology. Moderate symptoms of depression were experienced. (Fig. 2).

### Patient #5

A 51-year-old female who awoke from her sleep 2 years ago with a drooping right lip commissure. She was treated with prednisone and valaciclovir for 10 days, and a repeat dose of prednisone for eight weeks. She received acupuncture treatment because of her frequent headaches with her palsy. Bell’s Palsy symptoms were present for 10 months with no improvement. Similarly, radiological studies were unremarkable She had a history of systemic sclerosis affecting her ankles that made ambulation painful. Mild to moderate symptoms of depression were experienced.

### Patient #6

A 58-year-old female with a history of surgery for a 3 cm. right-sided acoustic neuroma 8 years ago, and developed Bell’s Palsy 24 h postoperatively. Radiological studies were normal. Treatment consisted of physical therapy and botulinum toxin. No improvement was seen on this regimen. Her symptoms have been stable and unchanged for 8 years prior to entering the study. Mild to moderate symptoms of depression were experienced.

### Patient #7

A 55-year-old female developed a spontaneous left earache, headache, and epiphora 1 year ago. Immediately a left-sided Bell’s Palsy developed. After treatment with prednisone and valaciclovir for one week, she received vitamin B-12 shots, facial massage, and botulinum toxin with an improvement in her left-sided epiphora only. She had left-sided Bell’s Palsy for 11 months. Mild to moderate symptoms of depression were experienced.

## Timeline

### Initial visit

- ◦ Screen potential participants. Perform baseline assessments. Exam. Consent.
- ◦ Labs: CBC, CMP, U/A.
- ◦ Facial Disability Index and House-Brackmann scoring.
- ◦ Radiographical studies reviewed. If none is available or not performed, CT of the face is ordered.

### Week 1

- ◦ Ultrasound facial nerve/parotid.
- ◦ Administer 13 cc ExoFlo™ in 100 cc normal saline intravenously.
- ◦ Inject 2 cc ExoFlo™ directly into tissue around the facial nerve.

### Week 2

- ◦ Repeat of Week 1

### Week 3

- ◦ No intervention.

### Week 4

- ◦ Repeat of Week 1 and 2.
- ◦ Final Assessments
- ◦ Facial Disability Index and House-Brackmann scoring. Medical
- ◦ Re-Evaluation

### Week 5

- ◦ Zoom call to inquire

## Diagnostic assessment

In this study, all patients were examined by an independent physician prior to entering the study. All medical records were reviewed and additional studies were ordered as needed to ensure not an exhaustive workup, but a safe and reasonable standard of compliance, and a baseline to proceed with the study. All protocols were discussed for more detailed patient understanding, as well as informed consent details. All cases had been seen and treated by their personal physicians and had exhausted treatment options. The time since the onset of symptoms was different in each case, but the treatment protocol was the same in all cases. There were no excluded cases with the manifestation of facial palsy in the study as patient selection was thorough. At the time of entering the study, all patients were off Botox except for one (#7) who used it to control epiphora and was free of any other contributary modalities that could interfere with the study.

## Therapeutic intervention

Each patient, before treatment and upon completion of the study was scored with the House–Brackmann scale, as well as a Facial Disability Index score. EVs were administered as follows on the schedule outlined above in the Timeline section. ExoFlo™ in 100 cc’s normal saline intravenously with a synchronous injection of 2 cc’s ExoFlo™ directly into tissue around the facial nerve with ultrasound guidance.

## Results and outcomes

### Analysis

In this study, all patients were examined by an independent physician prior to entering the study. All medical records were reviewed and additional studies were ordered as needed to ensure not an exhaustive workup, but a safe and reasonable standard of compliance, and a baseline to proceed with the study. All protocols were discussed for more detailed patient understanding, as well as informed consent details. All cases had been seen and treated by their personal physicians and had exhausted treatment options. The time since the onset of symptoms was different in each case, but the treatment protocol was the same in all cases. There were no excluded cases with the manifestation of facial palsy in the study as patient selection was thorough.

**Fig. 3:**
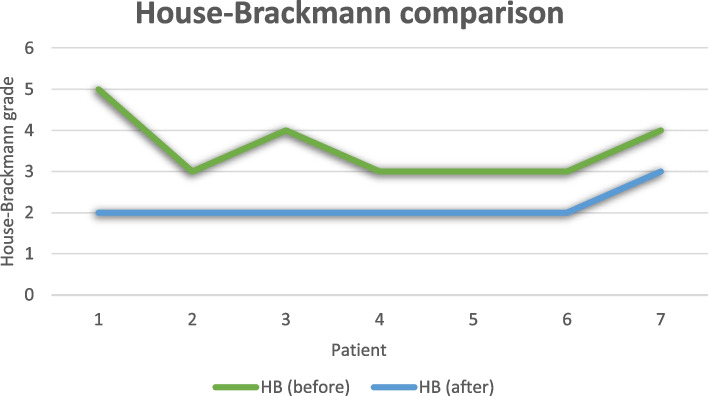
Comparison of House-Brackmann index grade before and after treatment for each patient

Each patient, before treatment and upon completion of the study was scored with the House–Brackmann scale, as well as a Facial Disability Index score (Table 1). Before treatment, cases appeared with severe and moderately severe states of paralysis, with a maximum House-Brackmann score of 5 and minimum score of 3. After treatment completion, there was a decrease of House–Brackmann scores in all patients (Fig. 3). They all decreased to a minimum House-Brackmann score of 2 except for one (Patient #7), displaying mild impairment of the disease, as Fig. 3 shows. House-Brackmann scores before and after treatment were analyzed statistically for the total number of patients using a paired t-test to determine whether, on average, there is a change between these two variables for the same subject. The statistic t-test = 4.804, with 6 degrees of freedom for a 95% confidence interval, and significance level a = 0.003, less than a = 0.05 shows there is significant evidence that the House-Brackmann index decreased after the treatment by an average of 1.429 (Table 3).

**Table 1:** Clinical assessment before and after treatment

Patient #	House-Brackmann	House-Brackmann	Facial Disability Index		Facial Disability Index		Adverse Events
	Before treatment	After treatment	Physical Index		Social Index		
			Before treatment	After treatment	Before treatment	After treatment	
1	V	II	44	71.5	48	79.2	None
2	III	II	77	88	56	76	None
3	IV	II	49.5	71.5	52	72	None
4	III	II	82.5	137	60	84	None
5	III	II	66	93.5	48.4	64	None
6	III	II	66	88	60	48	None
7	IV	III	77	110	44	44	None

**Fig. 4:**
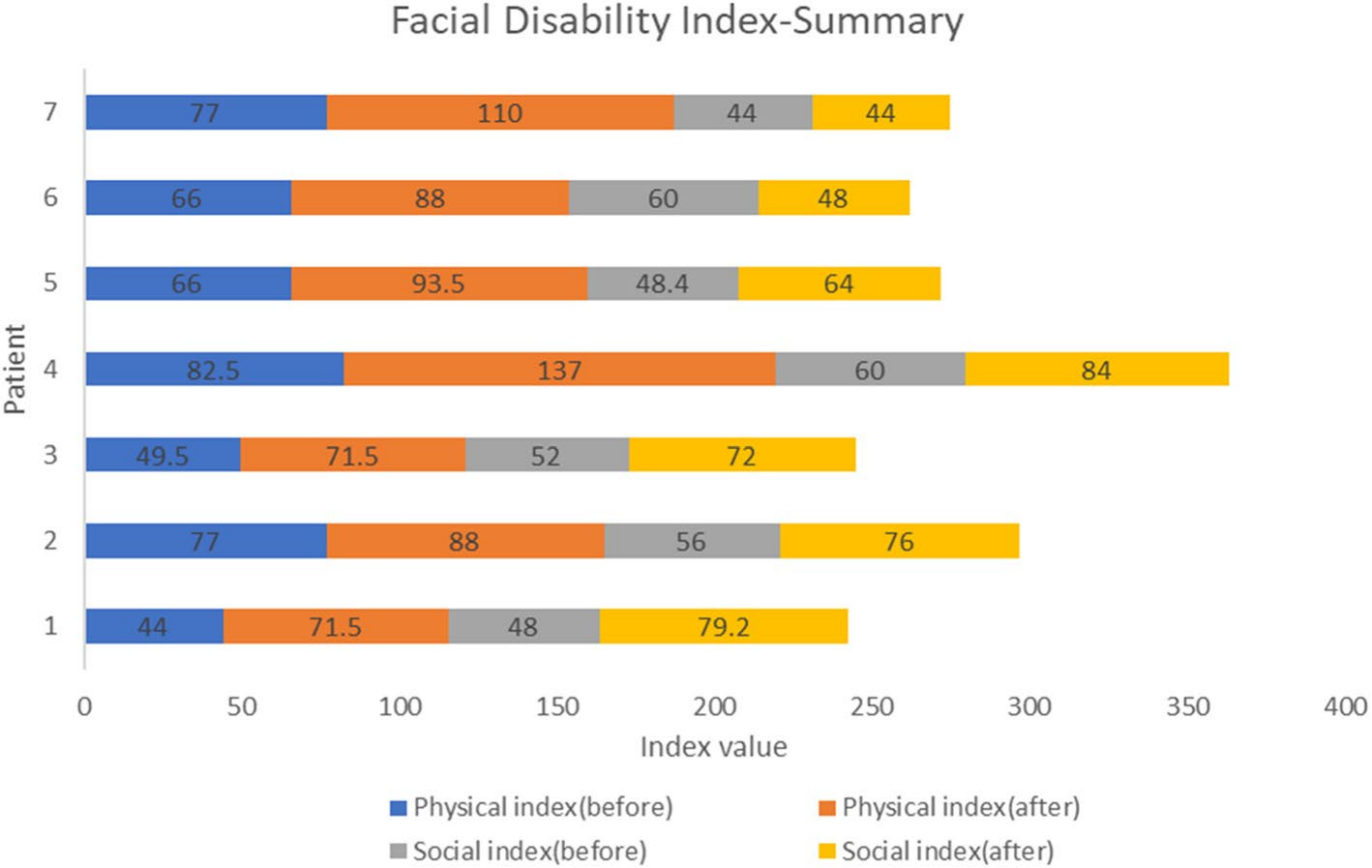
Summary of Facial Disability Index before and after the treatment with its two components physical and social index in each patient

The Facial Disability Index has two components, the Physical Index and Social Index, which were measured before and after the treatment for each patient as is shown in Fig. 4. Every patient scored a significant increase in Physical Index and Social Index values at the end of the treatment, except for one (patient #6). None of them have shown a decrease in functionality. The analyzed data for a total of 7 variables showed, that the Physical Index before the treatment was registered in a minimum value of 44, and after treatment reached a maximum value of 137 (Table 2). Also, a statistical analysis using a paired t-test was conducted to determine whether, on average, there was a change in Physical Index before and after the treatment. The statistic test t = -5.542, with 6 degrees of freedom for 95% confidence interval, and significance level a = 0.001, less than a = 0.05. This shows evidence of significant changes of Physical Index after the treatment, by an average of 28.21 (Table 3).

**Table 2:**
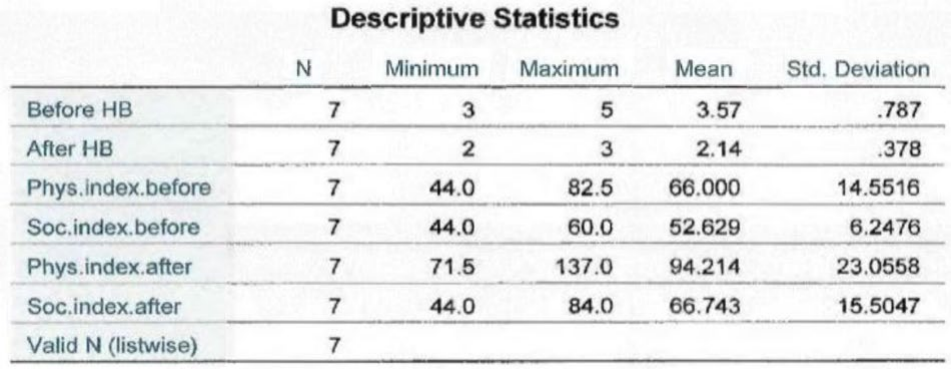
Analysis of descriptive statistics such as, mean values, minimum and maximum values for each parameter assessed in this study

**Table 3:**
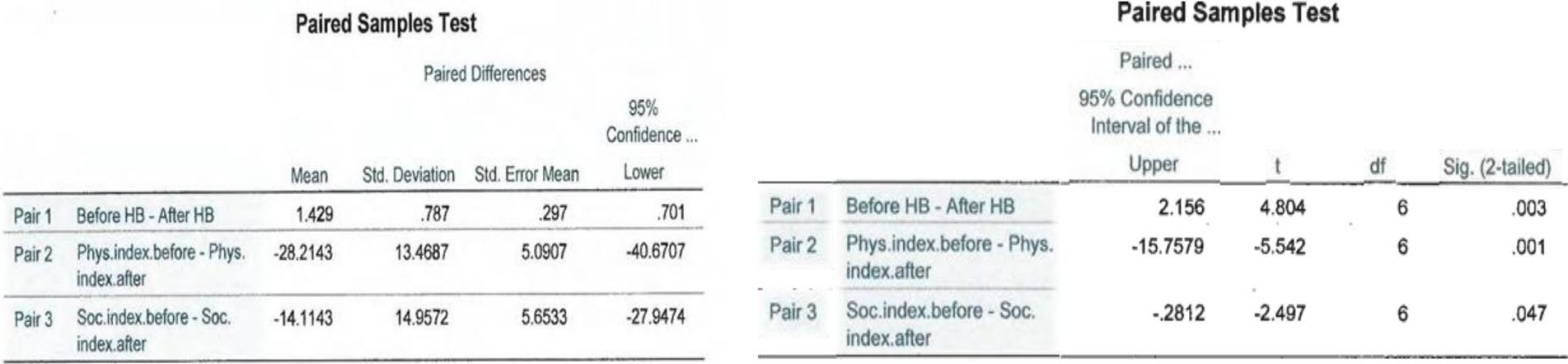
Statistical t-test values, and significance level values (2-tailed) for a 95% confidence interval are conducted for each pair of variables

The Social Index before treatment was scored at its minimum of 44, and after treatment, it had an increase to a maximum value of 84 (Table 2). A statistic-paired t-test was made to determine whether there was a change of this parameter before and after the treatment. The statistic test t = -2.497, with 6 degrees of freedom for a 95% confidence interval, and significance level a = 0.047, less than a = 0.05 shows significant changes in Social Index after the treatment, by an average of 14.11 (Table 3). After treatment, each of the patients noticed an increase in face functionality, where there was significant impairment prior to treatment. Their social exposure had a significant improvement, and they claimed to feel more confident and socially interactive.

**Fig. 5:**
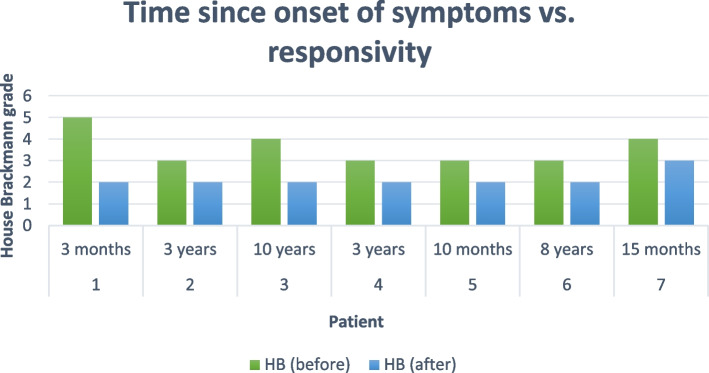
Comparison of time since onset of symptoms in each patient and their grade manifestation of disease versus responsivity of treatment

Data analysis after study completion showed a trend, and it is displayed in Fig. 5. Some patients manifested the disease symptomatology for eight to ten years. Others had them for several months. Although the subjects had different lengths of time since the onset of the symptoms and different House-Brackmann grade points, they all responded almost to the same point after treatment, respectively over a one-month period of time. The symptoms decreased from severe and moderately severe to mild.

The post-study Zoom calls revealed that all patients felt that they made significant improvements. They all desired to continue treatment and unanimously opined that the protocol was safely executed and posed no risks to their health. Consequently, they would unequivocally recommend this treatment to others with facial paralysis.

Patient #3 gained independent brow and eyelid motion, which he totally lacked prior to treatment. He also developed buccal control and gained overall facial expression. All patients developed a sequence of functional restoration beginning with the brow, then the eye, and finally the commissure.

## Discussion and conclusions

Patient safety concerns usually include medication errors, unsafe surgical care procedures, health-associated infections, safe injection practices, and diagnostic errors according to the World Health Organization ‘s website on patient safety [8]. As part of our IRB-approved protocol, we designed a sequence of events for our planned treatments, which were adhered to rigorously. Consequently, we did not experience any adverse events at all for the duration of our study. We demonstrated that a successful facial paralysis study was achievable, not only in a safe fashion by supervision but by strict adherence to protocols. Each patient was carefully monitored for medical progress, changes, and adverse events. Emergency access direct physician contact was available for each patient 24 h per day. Details were discussed with each patient in advance with emergency hospital backup facilities available. Independent medical evaluations were performed on all patients prior to entering the study.

All seven patients enrolled in the study improved with this treatment protocol. Four reported a sensation of nerve “tingling “, mild fasciculations, or “coming alive “of the nerve in the temple area, moving progressively to the buccal musculature, and to the angle of the mandible. Then a general relaxation was reported. Two patients reported the relaxation directly without fasciculations, as they did not feel the need to massage their faces every morning, as many patients do. After the second week of treatment, some patients developed independent motion of the affected eyelid – the restoration of the ability to wink. By the end of the second week into the third week, most developed early brow motion. Patients were allowed to rest on week 3, to allow the EVs an opportunity to work. They are generally thought to be effective in a treatment protocol for 4 weeks or more. After the third treatment, (week 4), commissure motion mildly improved. Although all patients began at different House-Brackman starting points, almost all ended at the same endpoint on the scale over the same period of time – four weeks.

I have previously reported a sentinel patient treated with mesenchymal stem cells for Bell’s Palsy with a similar outcome [1]. It is also interesting to note that the duration of symptomatology did not impact the final outcome in terms of the House-Brackman scoring.

Overall, 70–75% of facial paralysis cases are Bell’s Palsy, and 25–30% are secondary facial paralysis. Zhang published a detailed analysis of the etiology of Bell’s Palsy in 2019 [9]. He discussed the significance and implications of vascular secondary and tertiary ischemia causing the facial nerve sheath to thicken and inhibit nerve function [10].

Viral experience and involvement are well-established and documented in the literature. This includes Herpes simplex, Herpes zoster, Epstein-Barr virus, Coxsackie virus, rubella and cytomegalovirus, to name a few. Lyme disease had been recently added to this list [11]. Here in the case of patient #2, pregnancy was the cause. Demyelination, whether autoimmune or not, likely plays a role in the outcome of facial nerve pathology. High interleukins (IL-6) and (IL-8) are identified with elevated tumor necrosis factor (TNF-γ) and TNF- α [12].

In 1989 Liston and Kleid reviewed the known histopathology of Bell’s palsy cases in the literature [13]. Many reported cases had round inflammatory cells present in the facial nerves. Presenting their own patient, they reported inflammatory cells between the nerve bundles with normal blood vessels between the internal acoustic meatus and the stylomastoid foramen. They also reported a segmental breakdown of the myelin sheath. Demyelination may or may not be present. Dr. Sengupta published his findings on the effects of extracellular vesicles on COVID-19 cases [14]. EVs have been shown to be very effective against cytokine storms. We would expect to have an anti-inflammatory effect to diminish the inflammation of the facial nerve. But what we do not know from this study are the details of how remyelination proceeds from this intervention. Or, if there is a baseline EV communication between the neurons that plays a role in functional facial nerve restoration. We would expect to see myelin breakdown and chronic inflammation. There is conflicting information on T-helper cell levels. Gorodezky reported low CD4 cells [15]. Vedeler reported normal CD4 cell levels [16]. In reviewing Abramsky’s immune-mediated reports of myelin inflammation, Greco’s information would appear to support Liston and Kleid’s information on inflammation of the nerve [17, 18].

The length of time for nerve recovery may be dependent on the total extent of nerve damage present. However, partial nerve recovery is certainly achievable. Complete nerve recovery is within the realm of, not only possibility but probability.

One month was chosen as the length of time for the study for two reasons. First to see if any improvement could be made at all. The second reason was to see what would happen with the cessation of treatment. All patients responded positively with treatment. Recovery ceased with the cessation of treatment One could say that recovery was inevitable with the patients whose symptoms were three months duration or less. However, they too ceased advancement with the cessation of treatment. Thus, there is a pattern here. There were no complaints of bruising or pain. Inquiries were made at each patient encounter appropriately.

All patients involved in this study experienced long-term sequelae despite the length of time they sustained the symptomatology. Their quality of life was affected by the facial disfigurement. They all were depressed, felt alienated, and avoided meeting the public. Baugh recommended developing protocols to carefully evaluate facial paralysis patients and promote multidisciplinary guidelines with “expert consensus to resolve gaps in evidence [19].” Clearly, the duration of the treatment protocol needs to be longer than one month. The pathomechanism is still unknown. However, it appears that the mechanism is reversible. At last, these patients can have hope of a more effective, permanent, and low-risk treatment.

We have noted some interesting side effects. Patient #4 has a history of psoriatic arthritis that has been mildly problematic in the past. She has not experienced any psoriatic dermatopathology since her entry into the treatment protocol. This is not a surprising observation, as we have previously published the significant improvement of a psoriatic arthritis patient with stem cell therapy [20].

Patient #5 has a history of systemic sclerosis that primarily affected her ankles ambulating, and ascending stairs. Since her entry into the program, she has been completely pain-free and has been able to climb stairs without any discomfort. We did not feel that charting the above side effects was necessary, as this was a safety study. Randomized controlled trials (RCT) are the logical next step which would include these events, and usage of the eFACE Facial Nerve Clinician-Graded Scale would also be helpful in an RCT.

I firmly believe that if this study continued for three to six months, all patients would achieve House-Brachmann scores of 1. The patient’s age is the rate-limiting step. Again, not the duration of disease symptoms. All patients have held their improvements, without a return of severe symptomology. The only further therapy that would be required is lower the House-Brackmann scores further. Overall, extracellular vesicles have been found to be safe and effective in the treatment of facial paralysis.

## Patient perspective

The post-study Zoom calls revealed that all patients felt that they made significant improvements given the limitations of the study design and outcome measures. They all desired to continue treatment and unanimously opined that the protocol was safely executed and posed no risks to their health. Consequently, they would unequivocally recommend this treatment to others with facial paralysis.

## Follow-up

All patients continued to improve for a period of about 2 months after cessation of treatment. We saw this as further evidence that our future studies should continue longer than 1 month.

## Data Availability

Patient information and data are stored at the research facility in New York, listed in the correspondence address. Datasets are not public as they are being utilized in more advanced study projects ongoing as a result of this study as presented. We anticipate that they will become public in the near future. More information on the raw data is available from the senior author (PD).

## Abbreviations

EVs: Extracellular vesicles
Bell’s Palsy: Idiopathic Facial Paralysis
